# Identification of Blade Vibration Parameters Based on Improved Composite Reference Blade Tip-Timing Method

**DOI:** 10.3390/s25071955

**Published:** 2025-03-21

**Authors:** Liang Zhang, Xue Hu, Yitong Zheng, Wei Huang, Wenlong Yang

**Affiliations:** 1 Faculty of Mechanical Engineering and Automation, Liaoning University of Technology, Jinzhou 121001, China; 18342183655@163.com (X.H.); zhengyitong710@163.com (Y.Z.); 17375755194@163.com (W.H.); yyyangwenlong@163.com (W.Y.); 2 Key Laboratory of Vibration and Control of Aero-Propulsion System, Ministry of Education, Northeastern University, Shenyang 110819, China

**Keywords:** blade vibration, non-OPR BTT, instantaneous angular position, compound reference, straight line fitting

## Abstract

As the core component of an engine, the vibration characteristics of blades directly affect the stability and lifespan of the engine. Thus, monitoring the vibration status of the blades is essential. This paper presents an improvement on the blade tip timing (BTT) method based on Composite Reference. The conventional BTT method, when computing blade vibration displacement, typically presumes a constant rotor speed per revolution, which fails to account for the rotor’s instantaneous state conditions accurately. In this study, the state space equations for the rotor’s instantaneous angular velocity and position are derived and solved via a Kalman filter to rectify the rotor’s instantaneous speed. Furthermore, the composite reference method is improved by using the multi-sensor straight line fitting (SLF) method of all sensors, enhancing the precision of blade vibration displacement identification. The efficacy of this approach has been validated through simulation experiments.

## 1. Introduction

Blade condition monitoring is a key aspect in ensuring the healthy operation of aero-engines, especially when the engines face challenges such as higher rotational speeds, greater aerodynamic loads, and higher-temperature environments [1]. Through effective condition monitoring, the reliability of engine blades can be significantly improved, reducing the risk of potential failures. The application of this monitoring technology is crucial for maintaining and extending the service life of engines.

Currently, blade vibration monitoring methods are mainly divided into two categories: invasive and non-invasive. The invasive measurement method is primarily the strain gauge measurement method [2], which, although considered the most reliable experimental method, is not entirely suitable for modern advanced instrumentation needs due to its complex installation, potential to interfere with local fluid flow fields, and inability to function for long periods in extreme environments [3]. Non-invasive methods, such as BTT measurement methods, have become typical technologies for blade-disk vibration measurement in industrial equipment [4]. However, the type of sensors, the installation location of the sensors, and the data post-processing algorithms have not yet been standardized, requiring proof of reliability under different operating conditions [5].

In recent years, BTT data analysis methods have seen extensive development and application. Reinhardt et al. [6] analyzed timing errors in detail using OPR sensors and proposed a reference method to improve accuracy. Zhang et al. [7] proposed a new BTT method based on multiple reference phases, setting multiple reference phases on the shaft. Gallego-Garrido et al. [8] proposed a BTT method for multi-modal vibration. Liu et al. [9] proposed a BTT method based on reconstructed order analysis for blade vibration measurement under variable speed conditions. However, all these methods require key phase sensors as a reference to calculate blade vibration. The use of key phase sensors causes some problems in the practical application of BTT technology. First, in most cases, due to limited space for the engine, the installation of key phase sensors is very difficult. Second, if a key phase sensor fails or the signal is lost, then the entire BTT system will fail. Moreover, if a key phase sensor falls off, it can also damage the engine. Therefore, the ideal condition for the application of BTT technology is to eliminate the key phase sensor. To achieve this goal, researchers have made a lot of efforts.

In 2005, Michael Zielinski and Gerhard Ziller of MTU Germany were the first to propose the concept of keyless BTT measurement technology [10]. Russhard [11] proposed a method to generate a virtual reference signal through SLF. Later, Chen et al. [12] further developed the SLF method and proposed a new composite reference method. Guo et al. [13] used the installation position of the BTT sensor as a reference and calculated blade vibration and speed based on the Time of Arrival (TOA) signal. Fan et al. [14] used the difference in blade vibration displacement between two sensors to identify the vibration parameters of the blade. However, methods using blade or BTT sensors as a reference still face challenges in handling speed fluctuations and directly acquiring blade vibration displacement. Although the composite reference method can directly obtain the vibration displacement of the target blade, this method assumes that the rotor speed is constant within each revolution when calculating the speed, which is inaccurate for the instantaneous state of the rotor.

Therefore, this paper, based on the composite reference method, utilizes a multi-sensor SLF method [15] to more accurately determine the theoretical TOA of the reference blade, and employs a Kalman filter algorithm to determine the instantaneous rotational speed of the reference blade. By establishing the correlation between the vibration displacements of other blades and the reference blade, the vibration displacements of the remaining blades can be determined using the vibration displacement of the reference blade. This reduces the error in the measurement results of blade vibration displacement and improves the accuracy of blade vibration displacement identification.

## 2. SLF Method

When the blade rotates without vibration, the cumulative angle Φ swept by the blade at time t after sampling is described as follows:

(1) $$\Phi \left(t\right)=2\pi \left(\frac{1}{2}at^{2}+f_{0}t\right)$$

where *f*_0_ is the rotational speed frequency of the blade at the start of sampling, in Hz, and a is the acceleration, in Hz/s.

Since the blade vibrates during rotation, the cumulative angle Φ of the blade tip at time t after sampling can be expressed as follows:

(2) $$\Phi \left(t\right)=2\pi \left(\frac{1}{2}at^{2}+f_{0}t\right)+\Delta \Phi$$

**Figure 1:**
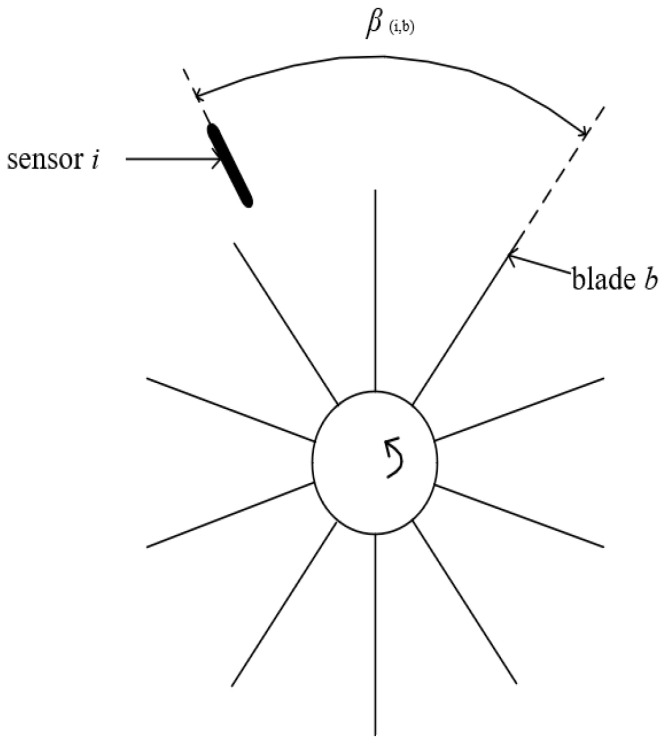
Diagram of blade rotation angle *β*.

In Formula (2), ΔΦ is the angle generated by blade vibration. As shown in Figure 1, when the blade b passes through sensor i without vibration, the sensor probe records the blade TOA, and the cumulative angle $\Phi_{\left(n,b\right)}^{i}$ of the blade at this time is described as follows:

(3) $$\Phi_{\left(n,b\right)}^{i}=n\cdot 2\pi +\beta_{\left(i,b\right)}\left(n=1,2,3\cdots \right)$$

where *β*_(i,b)_ represents the angle through which blade b rotates from the starting position to below sensor i.

*β*_(i,b)_ can be expressed as the following:

(4) $$\beta_{\left(i,b\right)}=\beta_{\left(1,1\right)}+\left(b-1\right)\frac{2\pi }{n_{b}}+\frac{\alpha_{\left(1,j\right)}}{360{}^{\circ}}2\pi$$

where *β*_(1,1)_ represents the angle between blade 1 and sensor 1, n_b_ is the number of blades, and *α*_(1,i)_ is the angle between sensor i and sensor 1.

In the keyless measurement method, when the first sensor probe records the first TOA of blade 1, sampling officially begins, so *β*_(1,1)_ = 0. If the blade does not vibrate during rotation, then the time the blade passes through the sensor is the theoretical TOA of the blade.

During the variable speed rotation of the blade (*a* ≠ 0 Hz/s), using the quadratic formula to solve for time t in Formula (1), the theoretical time $t_{t(n,b)}^{i}$ for blade b to reach sensor i on the nth revolution can be obtained:

(5) $$t_{t(n,b)}^{i}=\frac{-2\pi f_{0(n)}\pm \sqrt{4\pi f_{0(n)}^{2}+4\pi a_{\left(n\right)}\Phi_{\left(n,b\right)}^{i}}}{2\pi a_{\left(n\right)}}$$

Since $t_{t\left(n,b\right)}^{i}$ ≥ 0, the simplified Formula (5) is as follows:

(6) $$t_{t(n,b)}^{i}=-\frac{f_{0(n)}}{a_{\left(n\right)}}+\sqrt{{\left(\frac{f_{0(n)}}{a_{\left(n\right)}}\right)}^{2}+\frac{\Phi_{\left(n,b\right)}^{i}}{\pi a_{\left(n\right)}}}$$

During uniform speed rotation of the blade (a = 0), Formula (1) becomes the following:

(7) $$2\pi f_{0}t_{t}-\Phi =0$$

Then the theoretical time $t_{t(n,b)}^{i}$ for blade b to reach sensor i on the nth revolution is as follows:

(8) $$t_{t(n,b)}^{i}=\frac{\Phi_{\left(n,b\right)}^{i}}{2\pi f_{0(n)}}$$

During the data acquisition process, since the vibration value ΔΦ of the blade is extremely small compared to its cumulative angle Φ, the data points ($\Phi_{t(n,b)}^{i}$, $t_{t(n,b)}^{i}$) will inevitably fluctuate around the curve described by Formulas (6) or (8). Using the least squares method to fit the acquired data points ($\Phi_{t(n,b)}^{i}$, $t_{t(n,b)}^{i}$), the theoretical $t_{t(n,b)}^{i}$ TOA can be obtained. However, because Formula (6) is more complex than the linear Formula (8) due to its non-linear characteristics, the time required for the non-SLF process far exceeds that of SLF. Therefore, the linear Formula (8) can be used as the target function for fitting first. As this method only fits the data from a single rotation of the blade, it aims to predict the theoretical TOA of the blade within that rotation. Therefore, its effectiveness relies on conditions where the rotational speed is relatively high and fluctuates minimally, and the speed difference between blades within a single rotation is not significant. Under these circumstances, the average rotational speed of a single rotation can be approximated as the rotational speed of each blade as it passes the sensor. Conversely, if the rotational speed fluctuates significantly or the speed difference between blades is considerable, the SLF error will have a substantial impact on the measurement results.

Formula (8) can be rewritten as follows:

(9) $$t_{fit(n,b)}^{i}=k_{fit(n)}\Phi_{\left(n,b\right)}^{i}+b_{fit(n)}$$

(10) $$k_{fit(n)}=\frac{1}{2\pi f_{\left(n\right)}}$$

where b_fit(n)_ is the correction coefficient. By fitting this straight line with the least squares method, t_fit(n)_ can be obtained, which can be considered the theoretical TOA. In the original SLF method, only the blade TOAs of sensor 1 were fitted to obtain the theoretical TOAs of sensor 1. On that basis, this method will fit the blade TOAs of all sensors to obtain the theoretical TOAs of all sensors. Formula (4) is used to calculate the angle *β* that each blade rotates from the initial position to different sensors.

(11) $$\beta =\left[\beta_{\left(1,1\right)}\cdots \beta_{\left(1,b\right)}{\;\;\beta }_{\left(2,1\right)}\cdots \beta_{\left(i,b\right)}\right]$$

Arranged according to the order in which the blades arrive at the sensors (i.e., the size of the angle *β*_(i,b)_ when the blade rotates to the sensor), the distribution of the angle *β* between the blades in the fitting interval and the sensors is as follows:

(12) $$\beta^{\prime }=\left[\beta_{\left(1,1\right)}\cdots \beta_{\left(i,K\right)}\cdots \beta_{\left(j,b\right)}\right]$$

In Formula (12), *β*_(i,K)_ represents the angle through which blade K at the midpoint of the fitting interval rotates from the initial position to sensor i, and *β*_(j,b)_ represents the angle through which the blade b at the farthest end of the fitting interval rotates from the initial position to the farthest sensor j. Then, perform independent fitting on the data in the fitting interval to obtain the theoretical TOA of each sensor’s blades.

The blade vibration value obtained according to the BTT principle is as follows:

(13) $$x_{\left(n,b\right)}^{i}=\left(t_{\left(n,b\right)}^{i}-t_{t(n,b)}^{i}\right)\cdot v_{\left(n\right)}\approx \left(t_{\left(n,b\right)}^{i}-t_{fit(n,b)}^{i}\right)\cdot v_{\left(n\right)}$$

where $v_{\left(n\right)}$ is the linear speed of blade n.

(14) $$v_{\left(n\right)}=2\pi f_{0(n)}r=\frac{2\pi r}{T_{\left(n\right)}}=\frac{2\pi r}{t_{\left(n+1,b\right)}-t_{\left(n,b\right)}}\approx \frac{r}{k_{fit(n)}}$$

Thus, the vibration signal of the SLF method is ultimately obtained.

From Formula (14), it can be seen that v_(n)_ is calculated through k_fit(n)_ or T_(n)_, which gives the average linear speed of the nth revolution, not the instantaneous speed when the blade reaches the sensor probe. This will affect the measurement accuracy of blade vibration displacement. Therefore, it is necessary to correct the blade’s rotational speed.

## 3. The Improved Composite Reference Method

In the least squares SLF, the midpoint of the fitting interval usually has the smallest error [16]. This characteristic is extremely important for the accuracy of the keyless BTT method. Its impact will be explained in the following simulations.

This method first selects the blade at the midpoint of the SLF area as the reference blade, uses the SLF method to obtain the theoretical TOA of the reference blade K reaching the probe of sensor i, and then corrects the blade speed to obtain the vibration displacement of the reference blade K.

Diamond et al. [17] derived the state space equations for the instantaneous angular velocity and instantaneous angular position of the shaft, and used the Kalman filter to solve and correct the instantaneous speed of the rotor. This paper refers to its method and applies it to keyless measurement. The state equations for the speed of two consecutive revolutions of the rotor are as follows:

(15) $$\frac{1}{2}f_{\left(n,K\right)}^{i}T_{\left(n,K\right)}^{i}+\frac{1}{2}f_{\left(n+1,K\right)}^{i}T_{\left(n,K\right)}^{i}=\Delta \Psi$$

where $f_{\left(n,K\right)}^{i}$ is the speed frequency of the reference blade K when it reaches sensor i on the nth revolution, and $f_{\left(n+1,K\right)}^{i}$ is the speed frequency on the (n + 1)th revolution. ΔΨ represents the number of revolutions passed as 1. $T_{\left(n,K\right)}^{i}$ can be expressed as follows:

(16) $$T_{\left(n,K\right)}^{i}=t_{fit(n+1,K)}^{i}-t_{fit(n,K)}^{i}$$

When blade b passes through probe i on the nth revolution, the vibration displacement $x_{K(n,b)}^{i}$ of blade b relative to the reference blade K is the following:

(17) $$x_{K(n,b)}^{i}=x_{\left(n,b\right)}^{i}-x_{\left(n,K\right)}^{i}$$

Based on Formula (13), Formula (17) can be expressed as follows:

(18) $$x_{K(n,b)}^{i}=\left({t_{\left(n,b\right)}^{i}-t}_{fit(n,b)}^{i}\right)\cdot v_{\left(n,b\right)}^{i}-\left({t_{\left(n,K\right)}^{i}-t}_{fit(n,K)}^{i}\right)\cdot v_{\left(n,K\right)}^{i}$$

**Figure 2:**
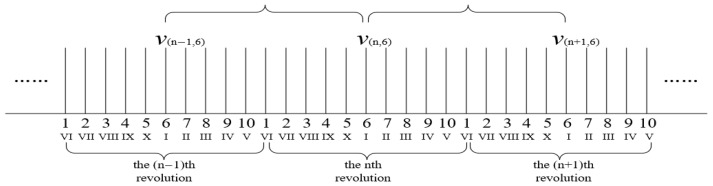
Diagram of blade speed solution.

The instantaneous speed of the reference blade K when it reaches the probe of sensor i can be obtained through the Kalman filter algorithm, and the instantaneous speed of the reference blade when it reaches the probe of sensor i of other blades can be obtained through the following method. As shown in Figure 2, taking a 10-blade example, taking blade 6 as the reference blade, since the rotor is performing uniform acceleration, the distance between adjacent speeds can be evenly divided into 10 parts, and through the instantaneous speed of the reference blade and the parts corresponding to each blade, the speed of each blade can be obtained, which can be considered as the instantaneous speed of other blades when they reach the probe of the sensor.

When b > K, the linear speed $v_{\left(n,b\right)}^{i}$ of blade b when it reaches sensor i on the nth revolution is as follows:

(19) $$v_{\left(n,b\right)}^{i}=v_{\left(n,K\right)}^{i}-\left(v_{\left(n,K\right)}^{i}-v_{\left(n+1,K\right)}^{i}\right)\frac{\left|K-b\right|}{n_{b}}$$

Since the calculation process when b < K is basically the same as that when b > K, to avoid verbosity in the main text, this paper omits the derivation of the calculation process for b < K.

Similarly, the instantaneous speed of the reference blade K when it reaches other sensors can also be obtained.

When *α*_i_ < *α*_j_, the linear speed $v_{\left(n,b\right)}^{j}$ of the reference blade K when it reaches sensor j on the nth revolution is as follows:

(20) $$v_{\left(n,b\right)}^{j}=v_{\left(n,K\right)}^{i}-\left(v_{\left(n,K\right)}^{i}-v_{\left(n+1,K\right)}^{i}\right)\frac{\left|\alpha_{i}-\alpha_{j}\right|}{360{}^{\circ}}$$

Similar to the above, this paper also omits the derivation of the calculation process for the case when α_i_ > α_j_.

When b > K, by substituting Formula (19) into Formula (18), we get the following:

(21) $$x_{K(n,b)}^{i}=\left(t_{\left(n,b\right)}^{i}-t_{\left(n,k\right)}^{i}\right)\cdot v_{\left(n,K\right)}^{i}-\left[\left(t_{fit(n,b)}^{i}-t_{fit(n,K)}^{i}\right)\cdot v_{\left(n,K\right)}^{i}+\left(t_{\left(n,b\right)}^{i}-t_{fit(n,b)}^{i}\right)\cdot \left(v_{\left(n,K\right)}^{i}-v_{\left(n+1,K\right)}^{i}\right)\frac{\left|K-b\right|}{n_{b}}\right]$$

The arc length L_(K,b)_ between blade b and the reference blade is the following:

(22) $$L_{\left(K,b\right)}=\frac{1}{2}v_{\left(n,b\right)}^{i}\left(t_{fit(n,b)}^{i}-t_{fit(n,K)}^{i}\right)+\frac{1}{2}v_{\left(n,K\right)}^{i}\left(t_{fit(n,b)}^{i}-t_{fit(n,K)}^{i}\right)$$

By substituting Formula (19) into Formula (22) and arranging it, we get the following:

(23) $$L_{\left(K,b\right)}=\left(t_{fit(n,b)}^{i}-t_{fit(n,K)}^{i}\right)v_{\left(n,K\right)}^{i}-\frac{1}{2}\left(t_{fit(n,b)}^{i}-t_{fit(n,K)}^{i}\right)\left(v_{\left(n,K\right)}^{i}-v_{\left(n+1,K\right)}^{i}\right)\frac{\left|K-b\right|}{n_{b}}$$

By substituting Formula (23) into Formula (21) and arranging it, we get the following:

(24) $$x_{K(n,b)}^{i}=\left(t_{\left(n,b\right)}^{i}-t_{\left(n,K\right)}^{i}\right)\cdot v_{\left(n,K\right)}^{i}-L_{\left(K,b\right)}-l$$

(25) $$l=\left(t_{\left(n,b\right)}^{i}-\frac{1}{2}t_{fit(n,b)}^{i}-\frac{1}{2}t_{fit(n,K)}^{i}\right)\left(v_{\left(n,K\right)}^{i}-v_{\left(n+1,K\right)}^{i}\right)\frac{\left|K-b\right|}{n_{b}}$$

From Formulas (17) and (24), we get the following:

(26) $$x_{\left(n,b\right)}^{i}=x_{\left(n,K\right)}^{i}+\left(t_{\left(n,b\right)}^{i}-t_{\left(n,K\right)}^{i}\right)\cdot v_{\left(n,K\right)}^{i}-L_{\left(K,b\right)}-l$$

Thus, the vibration displacement of all blades under sensor i can be obtained.

In general, a BTT monitoring system consists of multiple sensors. Similarly, based on the vibration conditions of each blade under sensor i and the installation angle of the sensor, the vibration conditions of each blade under other sensors can be calculated. The vibration displacement $x_{\left(n,K\right)}^{j}$ of the reference blade K when it passes through sensor j on the nth revolution is as follows:

(27) $$x_{\left(n,K\right)}^{j}=x_{\left(n,K\right)}^{i}+\left(t_{\left(n,K\right)}^{j}-t_{\left(n,K\right)}^{i}\right)\cdot v_{\left(n,K\right)}^{i}-L_{\left(i,j\right)}-L$$

(28) $$L_{\left(i,j\right)}=\frac{2\pi r\left(\alpha_{i}-\alpha_{j}\right)}{360{}^{\circ}}$$

When *α*_i_ < *α*_j_:

(29) $$L=\left(t_{\left(n,K\right)}^{j}-\frac{1}{2}t_{fit(n,K)}^{j}-\frac{1}{2}t_{fit(n,K)}^{i}\right)\left(v_{\left(n,K\right)}^{i}-v_{\left(n+1,K\right)}^{i}\right)\frac{\left|\alpha_{i}-\alpha_{j}\right|}{360{}^{\circ}}$$

From Formula (26), the vibration displacement $x_{\left(n,b\right)}^{j}$ of blade b when it passes through sensor j on the nth revolution is as follows:

(30) $$x_{\left(n,b\right)}^{j}=x_{\left(n,K\right)}^{j}+\left(t_{\left(n,b\right)}^{j}-t_{\left(n,K\right)}^{j}\right)\cdot v_{\left(n,K\right)}^{j}-L_{\left(K,b\right)}-l$$

In this way, the vibration displacement of blades under other sensors can be obtained.

## 4. Simulation

Blade vibration includes bending vibration, torsional vibration, and composite vibration, which are very complex and have an infinite number of degrees of freedom. This makes it difficult to accurately describe their motion with traditional mathematical formulas. Finite element analysis technology can solve this problem. It can not only deal with the complexity of the blades but also promote in-depth research on BTT analysis. The mathematical model of blade vibration is established using the finite element method.

### 4.1. Establishment of Simulation Model

The differential equation for the forced vibration of a multi-degree-of-freedom blade is the following:

(31) $$\left[m\right]\left\{\ddot{x}\right\}+\left[c\right]\left\{\dot{x}\right\}+\left[k\right]\left\{x\right\}=\left\{F\left(t\right)\right\}$$

where [m], [c], and [k] are the mass, damping, and stiffness matrices, respectively, {x} is the displacement during blade vibration, and {F(t)} is the external excitation force. The blade–disk system studied in this paper has ten blades, and each sector where a blade is located is simplified into a single-degree-of-freedom system model. [m], [c], and [k] are all 10 × 10 matrices, and {x} and {F(t)} are both 10-dimensional column vectors.

Under the condition of uniform acceleration of the blades and the presence of a single engine order, the excitation force acting on each blade is the following:

(32) $$F\left(t\right)=F_{A}cos\left(\pi N_{e}f_{v}+\varphi \right)$$

where F_A_ is the amplitude of the excitation force; N_e_ is the vibration engine order; *f*_v_ is the rotation speed frequency; *φ* is the initial phase.

**Figure 3:**
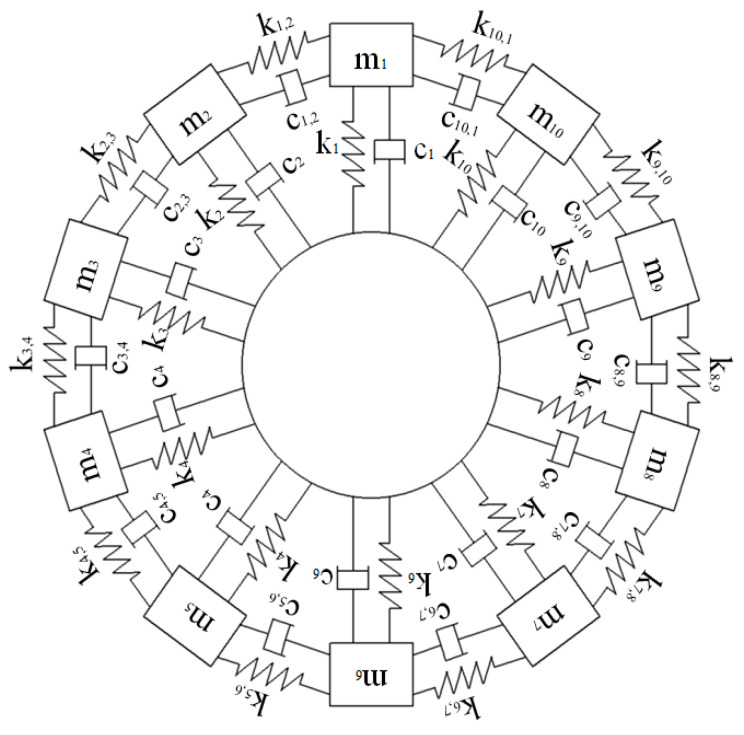
Ten-blade vibration system theoretical model.

Figure 3 is the theoretical model of the ten-blade vibration system. In practical applications, coupled vibration components between blades may exhibit negligible magnitudes under certain operating conditions. To facilitate the analysis of blade vibration characteristics and signal identification methodologies, the blade vibration model can be further simplified to an uncoupled single-degree-of-freedom (SDOF) system [18], i.e., $c_{1,2}=c_{2,3}=\cdots c_{9,10}=c_{10,1}=0,k_{1,2}=k_{2,3}=\cdots k_{9,10}=k_{10,1}=0$. The natural frequency of the blades is fn = 122.22 Hz, the mass of the blades is $m_{1}=m_{2}=\cdots m_{9}=m_{10}=0.5\;kg$, and the elastic coefficient of the blade material can be calculated $k_{1}=k_{2}=\cdots k_{9}=k_{10}=294,860\;N/m$ according to the formula: $f_{n}=\sqrt{k/m}/(2\pi )$. A damping coefficient of 0.005 is taken for the blade material, and the damping can be calculated as follows: $c_{1}=c_{2}=\cdots c_{9}=c_{10}=3.84\;N\cdot m/s$, according to the formula: $\xi =c/\left(2\sqrt{m\cdot k}\right)=0.005$. For convenience in subsequent analysis, in the simulation system, the vibration amplitude is set to 1 mm when the blades resonate, so the amplitude of the excitation force is as follows: $F_{A}=k\cdot 2\xi \cdot A=2.95\;N$. The number of sensors is five, with installation angles of 0°, 16°, 29°, 53°, and 75°, respectively. According to Formula (12), the midpoint blade of the fitting area is the 6th blade measured by the probe of sensor 2 and the 5th blade measured by the probe of sensor 4. This paper selects the former as the reference blade.

This paper will simulate the variable speed operation of the blades in two cases:

Case 1: Engine order Ne = 1, rotation speed frequency Ω increases from 110 Hz to 140 Hz, with an acceleration of 0.6 Hz/s.

Case 2: Engine order Ne = 2, rotation speed frequency Ω increases from 50 Hz to 68 Hz, with an acceleration of 0.6 Hz/s.

### 4.2. Speed Correction Results

**Figure 4:**
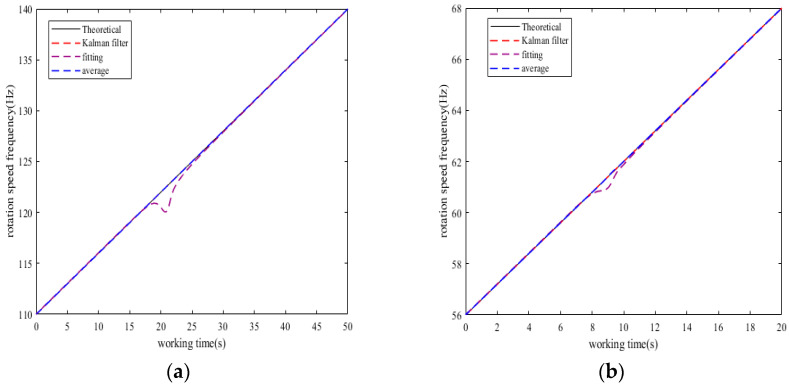
(**a**) Comparison of rotation speed frequencies in Case 1. (**b**) Comparison of rotation speed frequencies in Case 2.

**Figure 5:**
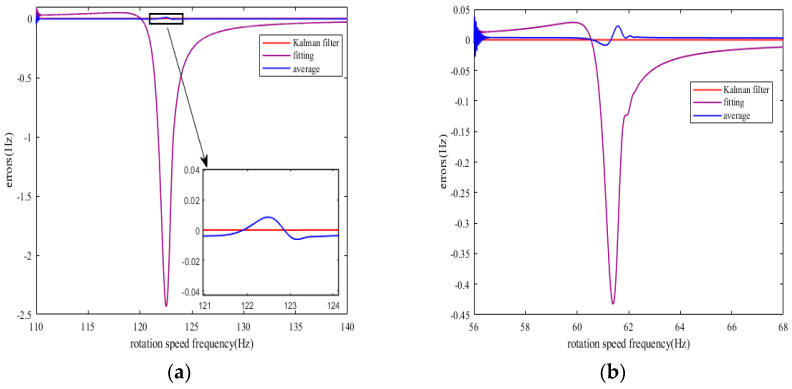
(**a**) Comparison of rotation speed frequency errors in Case 1. (**b**) Comparison of rotation speed frequency errors in Case 2.

Figure 4 is a comparison chart of the rotation speed frequency obtained by the three methods of Kalman filter algorithm, fitting method, and average value method with the theoretical rotation speed frequency for both cases. Figure 5 is a comparison chart of the error of the rotation speed frequency obtained by the three methods.

From Figure 4 and Figure 5, it is clear that the rotation speed frequency obtained by the improved method is superior in accuracy to the rotation speed frequency obtained by the fitting method and the average value method. The rotation speed frequency obtained by the Kalman filter algorithm basically coincides with the theoretical rotation speed frequency, which is more accurate compared to the average value method. However, the rotation speed frequency obtained by the fitting method has obvious differences from the theoretical rotation speed frequency during resonance, and this difference will cause errors in the calculation of the blade’s vibration displacement.

### 4.3. SLF Results

**Figure 6:**
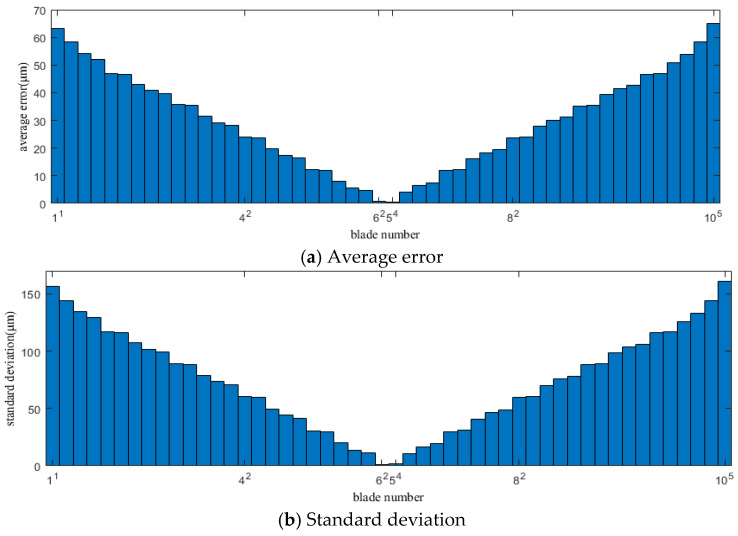
Average error and standard deviation of the vibration displacement calculation results of each blade within the fitting range in Case 1.

**Figure 7:**
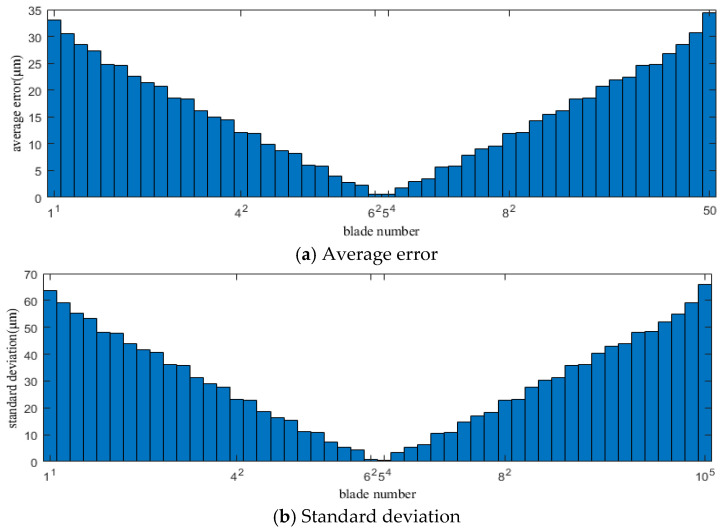
Average error and standard deviation of the vibration displacement calculation results of each blade within the fitting range in Case 2.

Figure 6 and Figure 7 show the average error and standard deviation between the vibration displacement of the blades measured by each sensor within the fitting range in Case 1 and Case 2 and the actual vibration displacement calculated by the SLF method.

In Figure 6 and Figure 7, it is clear that the average error and standard deviation for the vibration displacement of each blade obtained by the SLF method are different. Overall, the average error and standard deviation decrease from the first blade in the fitting range, reaching a minimum at the middle position of the fitting range, and then increase as the number of elements in the fitting area increases, reaching a maximum at the last blade in the fitting range, showing a clear convergence. This indicates that the blade at the midpoint of the fitting range has the smallest error and the least fluctuation. Therefore, the blade corresponding to or close to the midpoint of the fitting range will be an ideal and reliable “static” reference.

In the above figure, the numbers on the abscissa are designed in such a way that the base number represents the blade number, and the superscript represents the sensor number. As shown in the figure, ‘10^5^’ indicates information from blade number 10, as measured by sensor number 5. The same applies to the subsequent figures.

### 4.4. Improved CR-BTT Results

**Figure 8:**
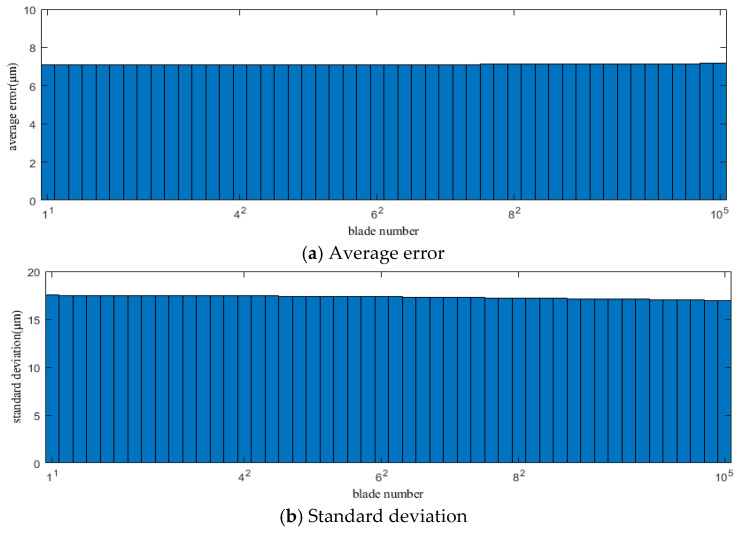
Average error and standard deviation of the calculation results for each blade using the original CR-BTT method in Case 1.

**Figure 9:**
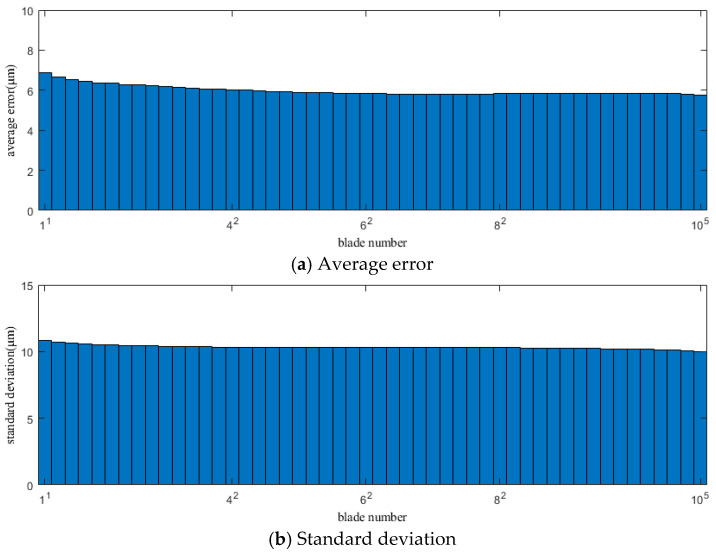
Average error and standard deviation of the calculation results for each blade using the original CR-BTT method in Case 2.

Figure 8 and Figure 9 show the average error and standard deviation of the calculation results for each blade using the original CR-BTT method under conditions 1 and 2, respectively.

**Figure 10:**
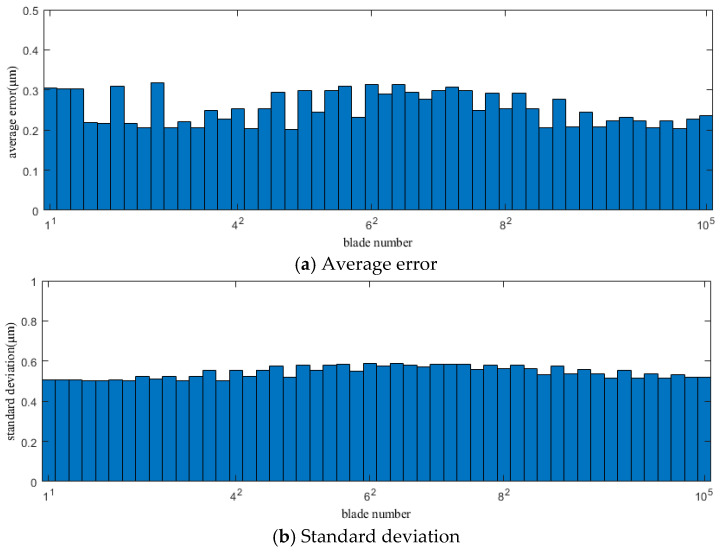
Average error and standard deviation of the calculation results for each blade using the improved CR-BTT method in Case 1.

**Figure 11:**
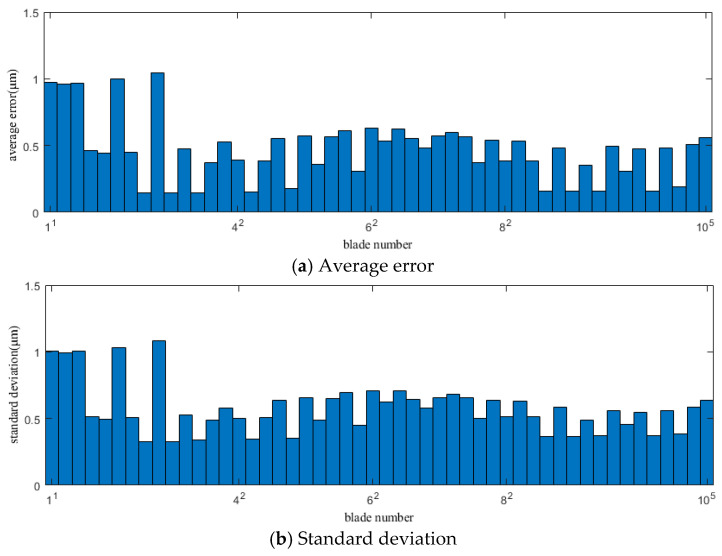
Average error and standard deviation of the calculation results for each blade using the improved CR-BTT method in Case 2.

Figure 10 and Figure 11 show the average error and standard deviation of the calculation results for each blade using the improved CR-BTT method under conditions 1 and 2, respectively.

**Figure 12:**
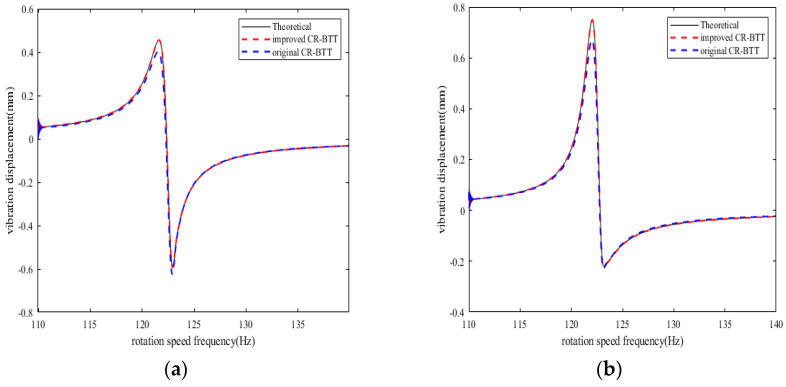
Blade vibration displacement diagram in Case 1. (**a**) Vibration displacement of blade 1 at sensor 1. (**b**) Vibration displacement of blade 10 at sensor 5.

**Figure 13:**
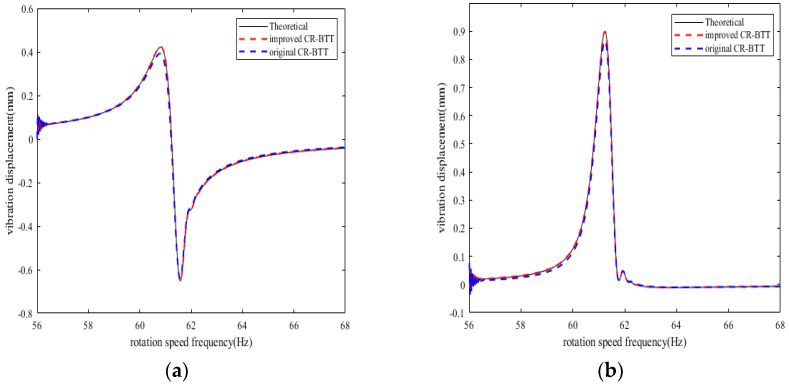
Blade vibration displacement diagram in Case 2. (**a**) Vibration displacement of blade 1 at sensor 1. (**b**) Vibration displacement of blade 10 at sensor 5.

From the results of Figure 8, Figure 9, Figure 10 and Figure 11, we can see that the improved CR-BTT method significantly reduces the average measurement error and standard deviation. To more intuitively compare the original CR-BTT method with the improved CR-BTT method, the vibration displacements of the blades at both ends of the fitting range obtained by the two methods are compared, as shown in Figure 12 and Figure 13. From Figure 12 and Figure 13, it is clear that the improved CR-BTT method can improve the accuracy of measuring blade vibration displacement. The original CR-BTT method has a certain error when measuring blade vibration displacement, and the error becomes more pronounced as the vibration value increases. The improved CR-BTT method measures blade vibration displacement, which basically coincides with the theoretical value.

## 5. Vibration Parameter Identification

To meet the practical measurement requirements that there are no more restrictions on the installation angle of the BTT sensors, a method of identifying synchronous vibration parameters of rotating blades with variable speed using multiple BTT sensors at arbitrary angles is adopted. At least five BTT sensors are required, and the specific parameter identification process is as follows.

The blade bending vibration displacement fitting curve equation [19] is the following:

(33) $$x=\frac{AQ}{\left(\frac{f_{v}}{f_{z}}\right)}\cdot \frac{\eta^{\prime }\cdot cos\varphi_{s}+sin\varphi_{s}}{\left(1+{\eta^{\prime }}^{2}\right)}+C$$

where A is the displacement caused by the amplitude of the external force, Q is the quality factor, *f*_z_ is the blade vibration frequency, and *f*_v_ is the blade rotation speed frequency. Also:

(34) $$\eta^{\prime }=\frac{1}{\eta }=Q\frac{1-{\left(\frac{f_{v}}{f_{z}}\right)}^{2}}{\frac{f_{v}}{f_{z}}}$$

(35) $$\varphi_{s}=N_{e}\alpha +\varphi_{0}$$

In Formula (35), *N*_e_ is the vibration multiple frequency, α is the installation angle of the sensor probe, and $\varphi_{0}$ is the initial phase.

The unknown parameters in Equation (33) can be written in vector form:

(36) $$\hat{a}={\left(A,f_{z},Q,\varphi_{s},C\right)}^{T}$$

Using the nonlinear function *x*$\left(f_{v},\hat{a}\right)$ of Equation (33) to represent the minimum square approximation fitting data, $\left({x_{j},f}_{vj}\right)$ of *x*$\left(f_{v},\hat{a}\right)$ is used to determine $\hat{a}$, through the Levenberg-Marquardt algorithm (L-M method) for nonlinear least squares data fitting [20]. From the L-M method fitting results, the resonance amplitude *A*_max_, center rotation speed frequency *f*_z_, quality factor Q, initial phase $\varphi_{s}$, and constant bias C can be obtained.

**Figure 14:**
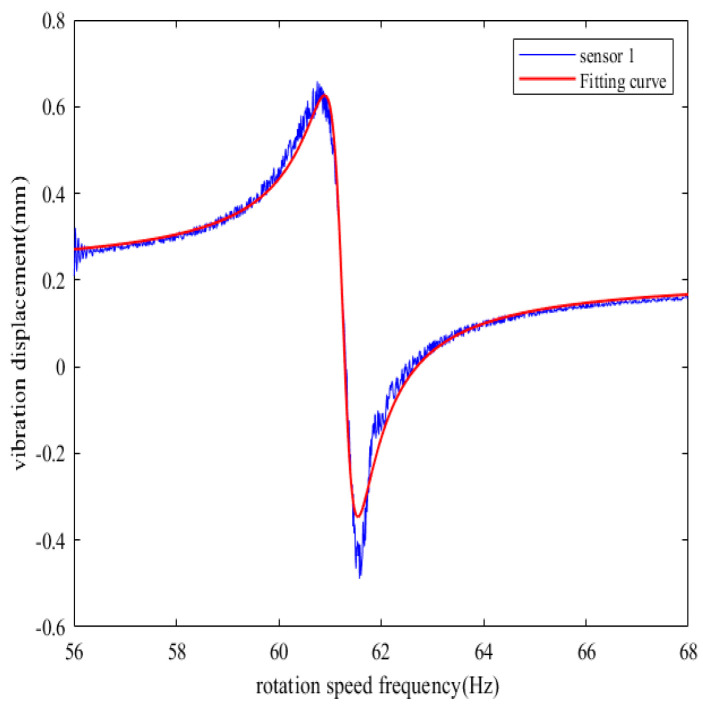
Fitting curve of synchronous vibration displacement of reference blade K measured by sensor 1 under variable speed.

If the rotation speed frequency increases linearly from 56 Hz to 68 Hz, the vibration multiple is 2, the synchronous resonance amplitude is 1 mm, the constant bias is 0.2 mm, and other system parameters remain unchanged, the simulation time is 20 s, and a 10% random noise is introduced into the measurement results of each BTT sensor. After processing the simulation data, Figure 14 is the fitting curve of the synchronous vibration displacement of the reference blade K measured by sensor 1 under variable speed.

By using the L-M algorithm in the nonlinear least squares method to fit the data obtained from the simulation, five vibration parameters are calculated: *A*_max_ = 1.02 mm, *f*_z_ = 61.271 Hz, *Q* = 95.387, $\varphi_{s}\;$= −8.648°, *C* = 0.211 mm, which basically remain consistent with the simulation parameters mentioned above.

To determine the vibration multiple of the twisted blade and accurately calculate the natural frequency of the blade, the relationship between the measured vibration phase difference and the installation angle of the sensor can be used to calculate the vibration multiple and the natural frequency of the blade by using multiple BTT sensors.

Taking sensor 1 as the reference standard, n BTT sensors are installed at angles α_1_, α_2_, …, α_n_ on the casing at the top of the blade. The difference between the vibration phase values of reference blade K measured by each BTT sensor and the vibration phase value of reference blade K measured by sensor 1 is the following:

(37) $${\Delta \varphi }_{i}=N_{e}\alpha_{i}$$

The phase difference value $\Delta \varphi_{i}$ between each BTT sensor and sensor 1 can be calculated by curve fitting, and integrated into [0°, 360°), the phase difference value $\Delta \varphi_{i}$ is represented by a vector as:

(38) $$\Delta \Phi =\left[{\Delta \varphi }_{1}\;\;\;{\Delta \varphi }_{2}\;\;\;\cdots \;\;\;{\Delta \varphi }_{i}\right]$$

If the actual vibration multiple of the blade is $N_{e}^{*}$, substitute the values that $N_{e}^{k}$ can take within a certain range into Equation (37) using an exhaustive algorithm, and integrate into [0°, 360°), the corresponding phase difference value $\Delta \varphi_{ik}$ represented by a vector is as follows:

(39) $$\Delta \Phi_{k}=\left[{\Delta \varphi }_{1k}\;\;\;{\Delta \varphi }_{2k}\;\;\;\cdots \;\;\;{\Delta \varphi }_{ik}\right]$$

The error between the phase difference value obtained by the exhaustive calculation and the actual measured phase difference value is *E*_k_, which can be represented as follows:

(40) $$E_{k}=\Delta \Phi_{k}-\Delta \Phi$$

where $E_{k}=\left[e_{1k}\;e_{2k}\;\cdots \;e_{ik}\right]$.

The magnitude of the phase difference value obtained by the exhaustive calculation deviates from the actual measured phase difference value, which is represented as follows:

(41) $$S_{k}=\sqrt{\frac{\sum_{i=1}^{n}e_{ik}^{2}}{n}}$$

Since the error cannot be completely avoided, the smallest *S*_k_ corresponding to exhaustive multiple $N_{e}^{k}$ is taken as the actual multiple.

**Figure 15:**
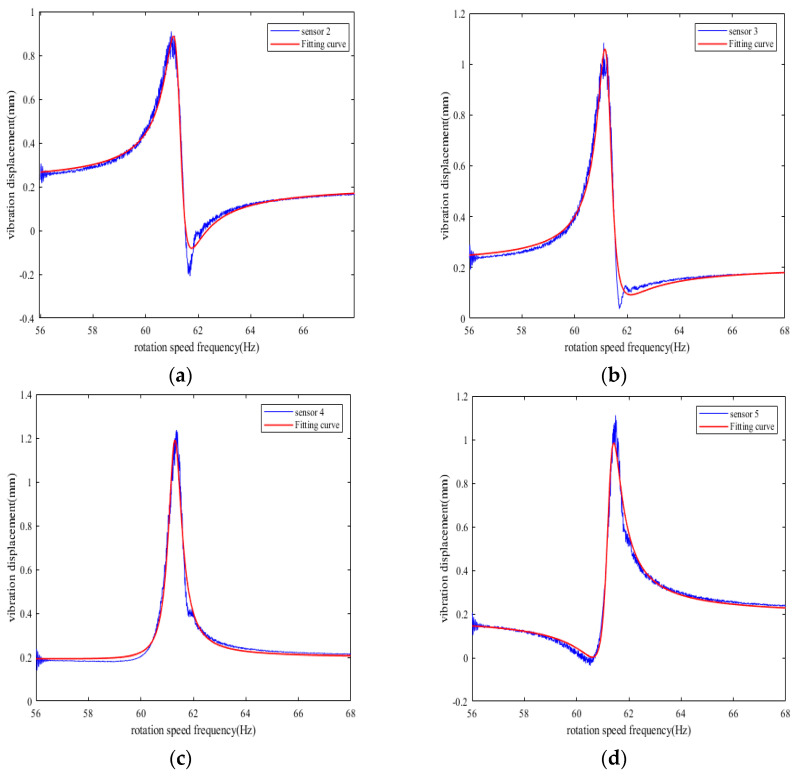
Fitting curve of synchronous vibration displacement of reference blade K measured by other sensors under variable speed. (**a**) Fitting curve of sensor 2. (**b**) Fitting curve of sensor 3. (**c**) Fitting curve of sensor 4. (**d**) Fitting curve of sensor 5.

Five BTT sensors are used for simulation analysis, with sensors 2–5 taking sensor 1 as the reference, and the installation angles are in sequence as follows: 0°, 16°, 29°, 53°, 75°, and a 10% random noise is introduced into the measurement results of each BTT sensor. After processing the simulation data, the fitting curves of the synchronous vibration displacement of reference blade K measured by each BTT sensor under variable speed can be obtained, as shown in Figure 15.

**Figure 16:**
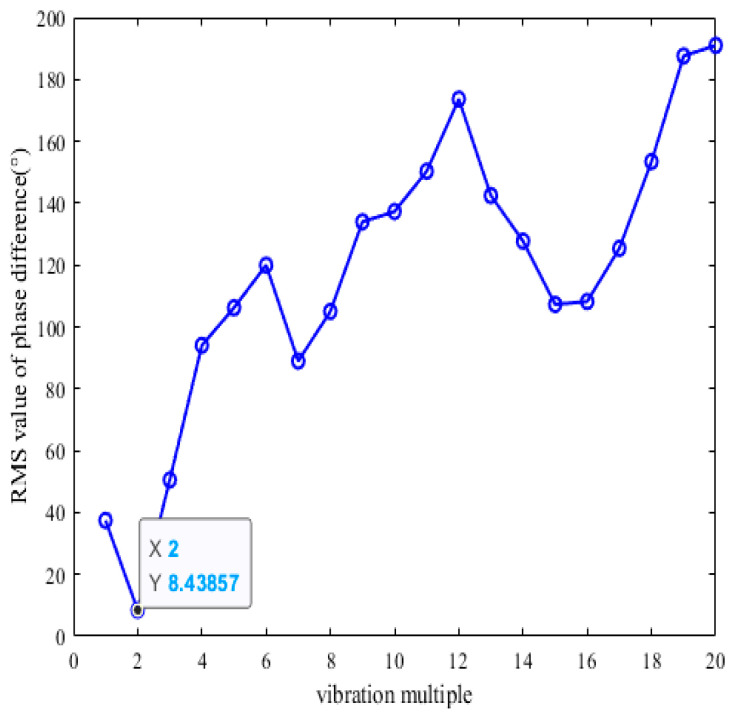
Calculation results of vibration multiple of reference blade K by exhaustive method.

The numbers are shown in Table 1. After calculation of vibration multiples from 1 to 20 by exhaustive calculation, Figure 16 shows the traversal results. From Figure 16, it can be seen that *N*_e_ = 2, the minimum root mean square value *S*_k_ = 8.44°. From Table 1, it can be seen from the results of identifying the synchronous vibration parameters of the reference blade K under the variable speed sweep that the center rotational speed frequency *f*_z_ = 61.271 Hz, the intrinsic frequency $f_{n}=2\times 61.271=122.542Hz$ of the blade, and the identifying frequency of the reference blade K, 122.542 Hz, have an error of only 0.322 Hz with the assumed frequency of 122.22 Hz for the simulation. Therefore, Five BTT sensors are mounted at any angle on the blade tip magazine, allowing accurate identification of the synchronized vibration parameters of torsionally angled blades under variable speed sweeps. The feasibility of the keyless-phase tip timing vibration measurement technique for identifying the vibration parameters of the torsion angle blade under variable speed sweep is theoretically verified.

## 6. Conclusions

This paper conducts theoretical derivation and analysis based on the keyless CR-BTT measurement method, and makes improvements by adjusting the fitting interval and correcting the instantaneous rotation speed of the reference blade. The effectiveness of the improved CR-BTT method is verified through simulation, and the accuracy of blade vibration displacement measurement is improved compared with the CR-BTT method. The results measured by the improved CR-BTT method basically coincide with the actual vibration values, while the original CR-BTT method has significant differences with the actual values when measuring at resonance.

At the same time, the method of identifying synchronous vibration parameters of rotating twisted blades under variable speed using multiple BTT sensors at arbitrary angles is adopted through simulation, which verifies that this method can accurately identify the synchronous vibration parameters of twisted blades under variable speed sweep frequency. Theoretically, the feasibility of keyless BTT vibration measurement technology for identifying blade vibration parameters under variable speed is verified.

## Figures and Tables

**Table 1:** Identification results of synchronous vibration parameters of reference blade K under variable speeds.

Sensor	*f*_z_ (Hz)	*A*_max_ (mm)	*Q*	*C* (mm)	ΔΦ (°)	ΔΦk (°)
sensor 1	61.271	1.02	95.387	0.211	0	0
sensor 2	61.269	1.01	96.133	0.209	31.971	32
sensor 3	61.272	1.01	96.222	0.207	58.199	58
sensor 4	61.276	1.01	98.488	0.199	106.528	106
sensor 5	61.269	1.02	95.863	0.192	150.363	150
mean	61.271	1.014	96.419	0.204	\	\

## Data Availability

The data used to support the findings of this paper are available from the corresponding author upon request. The data are not publicly available due to privacy.
